# A new method for estimating under-recruitment of a patient registry: a case study with the Ohio Registry of Amyotrophic Lateral Sclerosis

**DOI:** 10.1038/s41598-022-18944-9

**Published:** 2022-08-30

**Authors:** Meifang Li, Xun Shi, Jiang Gui, Chao Song, Angeline S. Andrew, Erik P. Pioro, Elijah W. Stommel, Maeve Tischbein, Walter G. Bradley

**Affiliations:** 1 Department of Geography, Dartmouth College, Hanover, NH 03755 USA; 2 Department of Biomedical Data Science, Geisel School of Medicine at Dartmouth, Hanover, NH 03755 USA; 3 HEOA Group, West China School of Public Health and West China Fourth Hospital, Sichuan University, Chengdu City, Sichuan Province 610093 China; 4 Department of Neurology, Geisel School of Medicine, Dartmouth-Hitchcock Medical Center, Lebanon, NH 03756 USA; 5 Section of ALS and Related Disorders, Cleveland Clinic, Cleveland, OH 44195 USA; 6 Department of Neurology, Miller School of Medicine, University of Miami, Miami, FL 33136 USA

**Keywords:** Amyotrophic lateral sclerosis, Data integration, Data acquisition, Data publication and archiving, Quality control, Statistical methods

## Abstract

We developed a disease registry to collect all incident amyotrophic lateral sclerosis (ALS) cases diagnosed during 2016–2018 in Ohio. Due to incomplete case ascertainment and limitations of the traditional capture-recapture method, we proposed a new method to estimate the number of cases not recruited by the Registry and their spatial distribution. Specifically, we employed three statistical methods to identify *reference counties* with normal case-population relationships to build a Poisson regression model for estimating case counts in *target counties* that potentially have unrecruited cases. Then, we conducted spatial smoothing to adjust outliers locally. We validated the estimates with ALS mortality data. We estimated that 119 total cases (95% CI [109, 130]) were not recruited, including 36 females (95% CI [31, 41]) and 83 males (95% CI [74, 99]), and were distributed unevenly across the state. For *target counties*, including estimated unrecruited cases increased the correlation between the case count and mortality count from *r* = 0.8494 to 0.9585 for the total, from 0.7573 to 0.8270 for females, and from 0.6862 to 0.9292 for males. The advantage of this method in the spatial perspective makes it an alternative to capture-recapture for estimating cases missed by disease registries.

## Introduction

Amyotrophic lateral sclerosis (ALS) is a progressive and fatal neuromuscular disease, with a lifetime risk of about 1:400^1^. The global incidence of ALS for all ages is estimated to be 0.78 per 100,000 person-years^2^. The age- and/or sex-standardized rates reported in Ireland, the United Kingdom, Italy, North America, and New Zealand have been reported to be 1.7–1.81^3,4^. The incidence rates in Asia, South America, and the Caribbean are lower^4^. Such differences offer insights into etiological factors^5^.

We developed a population-based ALS Registry for the US state of Ohio to determine the incidence of ALS in the state, the distribution and demographics of patients, and associations between ALS and environmental toxins/toxicants. We attempted to collect every newly diagnosed (incident) ALS patient in Ohio during a two-year index period from October 2016 through September 2018^6^. The completeness of the Registry is fundamental to achieving the aforementioned goals^7^. However, we were unable to trace every ALS case for at least two reasons: (1) not all centers/physicians had developed collaborative agreements with the Registry, and (2) patients from some parts of Ohio may have gone to other states for medical care.

Under-ascertainment is common in surveillance data collection^8^, in part because the U.S. lacks a mandatory reporting infrastructure for ALS disease^5^. Nelson et al. estimated that the completeness of ALS cases collected by the US national administrative healthcare databases was 76% for the years 2002–2004^9^, and Kaye et al. implied that the National ALS Registry of the US may have missed more than 40% of cases^10^. Witte et al. found that in the state of Georgia four data sources combined may have collected 90.7% of cases^11^. Jordan et al. used the death certificate data to estimate that in Baltimore and Philadelphia more than 11% of ALS cases had been missed during data collection efforts in those cities^12^. Preux et al. used three sources in Limousin (France) for the period 1994–1995 and estimated completeness of 65.7%^13^. Uenal et al. estimated a missing rate to be 18.9% in the ALS registry for Swabia (Germany)^14^, consistent with the claim of Rosenbohm et al. that for Southern Germany the clinical-epidemiological registry had collected 80% cases^15^.

Considerable effort has been made to estimate the number of unrecruited cases in ALS registries. The dominant method in literature is the capture-recapture process^1,9,11,13–18^. The basic idea of the capture-recapture method is to build statistical models based on the overlap between different sources and use these models to estimate the total number of cases, and hence the number of unrecruited cases. A fundamental assumption of this method is that the cases from each data source are random samples of all cases. Also, this method seems to be most applicable to situations with only a few data sources that are all covering the same base population. Another limitation of the method is that it only produces the total number of cases, without the spatial distribution of those cases.

These assumptions and limitations make the capture-recapture method unsuitable for our needs. First, our only data source was to identify individual institutions, which each primarily cover a distinct patient population from a given city or region, and thus are not a random sample of all ALS cases in Ohio. We only achieved collaborative agreements from a subset of the 20 + institutions in Ohio that may have diagnosed ALS cases. Second, our future objectives are to identify disease hotspots and detect the disease-environment associations, for which the spatial distribution of unrecruited cases, besides the total number, is fundamental and crucial.

Therefore, we developed a new method for estimating the distribution of unrecruited cases. Different from the capture-recapture method, our method is entirely based on local data, not requiring assumptions regarding the sampling of cases. Our method takes the population of each county as the only variable, noting that the county-level population data is easy to obtain. Our method directly estimates the number of unrecruited cases for each county, which not only can be summed up to get the total number of unrecruited cases for the entire state, but also allows us to assess the spatial association between the ALS disease and environmental factors.

## Results

### Statistically estimate the number of unrecruited ALS cases in Ohio during 2016–2018

The statistical estimation gave the *expected* case count for each *targeted county*. If the modeled *expected* case count > the number of recruited cases, then the difference is the estimated number of unrecruited cases. The total number of unrecruited cases from this phase of estimation is 35 (female) + 81 (male) = 116 (Table 1).

**Table 1:** Statistically estimated number of unrecruited ALS cases in Ohio during 2016–2018.

Sex	Number of recruited cases	Number of expected cases	Estimated number of unrecruited cases	95% CI of the estimated number of unrecruited cases
Female	128	163.08	35.08	[30.34, 39.82]
Male	155	236.20	81.20	[71.88, 96.58]
Total	283	399.28	116.28	[105.85, 126.81]

The output from the Poisson regression model contains decimal digits.

### Spatially adjusted number of unrecruited ALS cases in Ohio during 2016–2018

For both female and male cases, the local *Moran’s I* analysis identified one *LH* county, i.e., a county with a low incidence rate surrounded by counties with high rates. The smoothing process added one unrecruited case to the female case count and two to the male case count for that *LH* county. After these adjustments, the final estimated number of unrecruited cases in Ohio is 36 for females, 83 for males, and 119 in total (Table 2).

**Table 2:** Final estimated cases unrecruited by the Ohio ALS Registry during 2016–2018.

Sex	Number of recruited cases in the *target counties*	Number of recruited cases in all 88 counties	Estimated number of unrecruited cases	Rounded integer	% in the total number of cases in the *target counties*	% in the total number of cases in all 88 counites
Female	10	128	35.54	36	78.26	21.95
Male	36	155	82.81	83	69.75	34.87
Total	46	283	118.35	119	72.12	29.60

“% in the total number of cases in the *target* counties” and “% in the total number of cases in all 88 counties” were calculated with the rounded integer number of unrecruited cases divided by the number of expected cases, where the number of expected cases equals to the sum of the number of recruited cases and unrecruited cases.

Table 2 shows that the number of unrecruited male cases is more than two times the number of unrecruited female cases. Unrecruited cases account for the majority of all cases in the *target counties* (78.26%, 69.75%, and 72.12% for females, males, and total, respectively). They also account for a substantial percentage in all cases statewide (21.95%, 34.87%, and 29.60% for females, males, and total, respectively).

**Figure 1:**
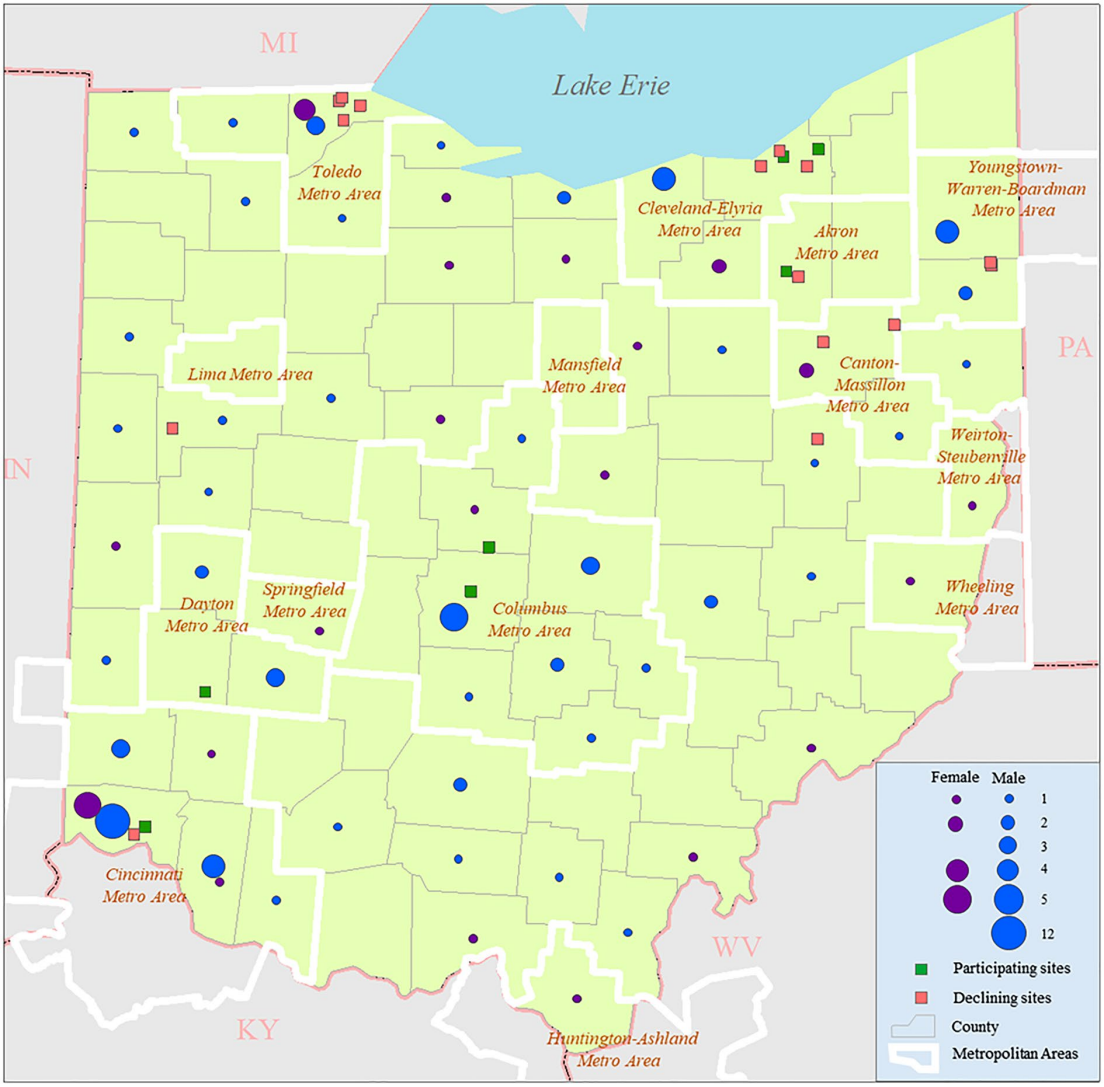
Estimated numbers of unrecruited ALS cases in individual Ohio counties.

**Figure 2:**
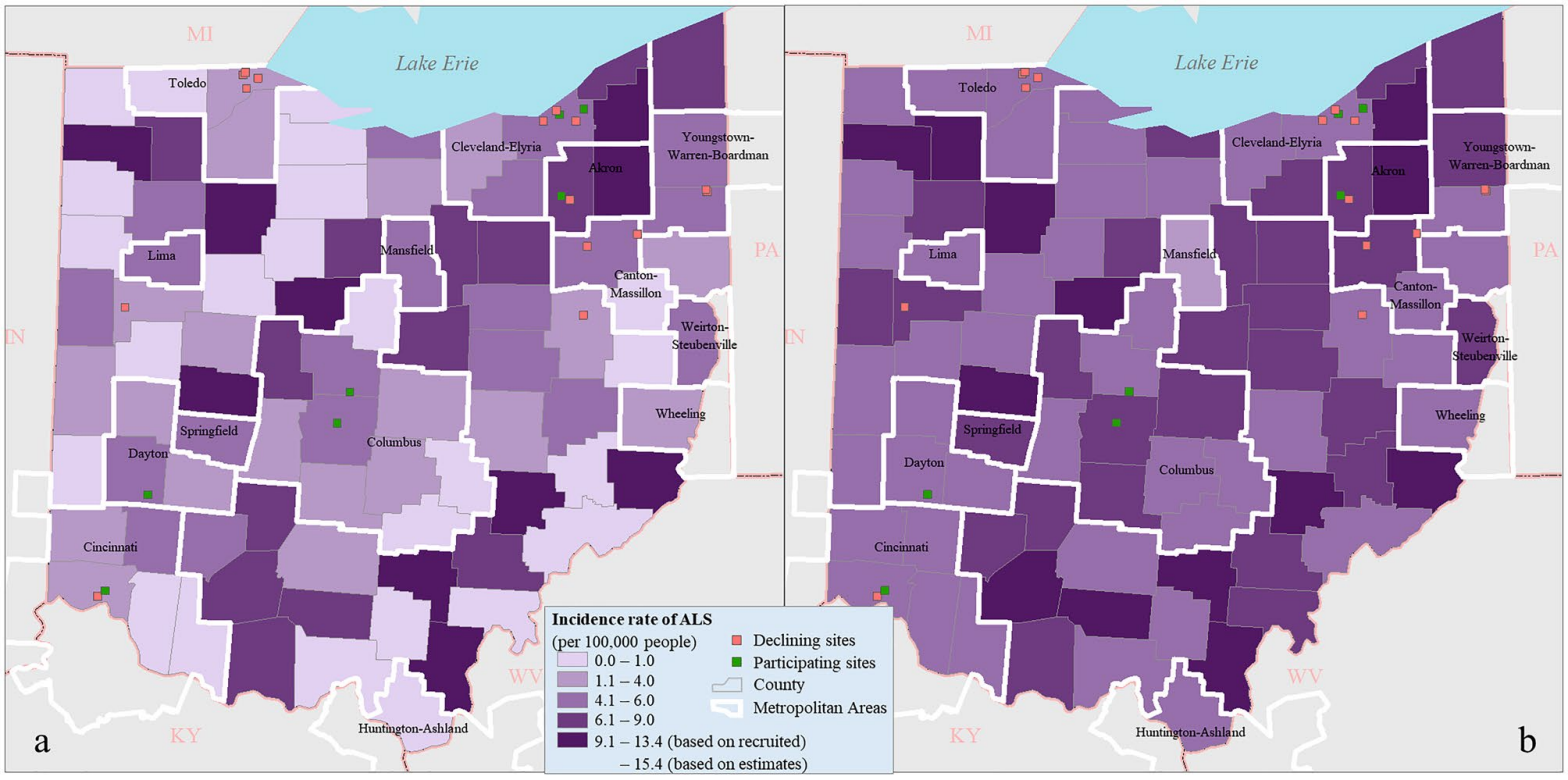
The incidence rate of ALS in Ohio. (**a**) Before including the estimated unrecruited cases; (**b**) after including the estimated unrecruited cases.

Figure 1 shows the spatial distribution of estimated unrecruited cases in Ohio. Figure 2 compares the county-level incidence rate based on recruited cases (left) to that based on the final compiled estimation results (right), thereby demonstrating the effect of the estimation.

### Validation of estimation results based on the mortality data

Table 3 lists Pearson’s correlation coefficient (*r*) values between the county-level incidence case count and the mortality case count.

**Table 3:** Pearson’s correlation coefficients (*r*) between county-level ALS case count and ALS mortality count in Ohio.

Sex	Non-target counties	All 88 counties without estimated unrecruited cases	All 88 counties with statistically estimated unrecruited cases	All 88 counties with spatially adjusted unrecruited cases	Target counties without estimated unrecruited cases	Target counties with statistically estimated unrecruited cases	Target counties with spatially adjusted unrecruited cases
Female	0.9114	0.8644	0.9022	0.9025	0.7573	0.8256	0.8270
Male	0.9395	0.8202	0.9341	0.9325	0.6862	0.9320	0.9292
Total	0.9666	0.9067	0.9668	0.9648	0.8494	0.9621	0.9585

First of all, for those counties that we assumed had all cases recruited (referred to as non-target counties in Table 3), including the *reference* counties and those counties that have even higher incidence rates based on their recruited cases, there is a strong correlation between the case count and mortality count (*r* = 0.9114, 0.9395, and 0.9666 for females, males, and total, respectively). These high Pearson’s correlation coefficient values provide validation of our statistical method of correcting the Registry for unrecruited cases.

In terms of matching the mortality count, the inclusion of the estimated unrecruited cases considerably improves the quality of the ALS case base. Between sexes, the improvement of the correlation for males is greater than that for females. When calculated with data from all 88 counties, the *r* of male cases increases from 0.8202 to 0.9325, while the correlation among females *r* increases from 0.8644 to 0.9025. When calculated only using the data of the *target* counties, which appeared to have low case ascertainment, the *r* of male cases increases from 0.6862 to 0.9292, compared with females’ 0.7573 to 0.8270. The increase of *r* for the *target* counties more indicatively demonstrates the improvement made by the unrecruited case estimation on the quality of the case base.

The spatial adjustment, while theoretically reasonable, did not considerably alter the results from the statistical estimation in the particular case of Ohio. The one female case added by the spatial adjustment slightly improved the *r* (from 0.9022 to 0.9025 for all counties, and from 0.8256 to 0.8270 for the *target* counties), whereas the two male cases added by the spatial adjustment eventually reduced the *r* to a small extent (from 0.9341 to 0.9325 for all counties, and from 0.9320 to 0.9292 for the *target* counties). When the female and male cases are put together, the overall *r* was slightly decreased by the spatial adjustment (from 0.9668 to 0.9648 for all counties, and from 0.9621 to 0.9585 for the *target* counties).

## Discussion

The Ohio ALS Registry aimed to construct a comprehensive case database of incident ALS for a given two-year time period, which is a fundamental step in the effort to identify hotspots of the disease and detect potential environmental risk factors of ALS in Ohio^6^. However, like many previous studies, we were not able to recruit every case, and the capture-recapture method was not suitable for our study design. Thus, we developed an alternative method to estimate the number of unrecruited cases and their spatial distribution. The results of our validation with the ALS mortality database for the state of Ohio are encouraging.

Our estimation indicates that, statewide, the unrecruited cases account for less than 30% of all cases for our two-year period, which is similar to the wide range of results of the previous studies^9–15^. We also found that the percentage of unrecruited male cases is higher than that of female cases (34.87% vs. 21.95%), which follows the base rates of the disease (with a global incidence rate of 1.9 in males and 1.3 in females)^19,20^, and is also qualitatively consistent with Nelson et al.^9^. The male predominance of unrecruited cases includes 44 veterans diagnosed in the Veterans Affairs (VA) system who were not able to be included in the Registry^6^. We found the number of unrecruited cases varies drastically from county to county in Ohio, and that spatial distribution also differs greatly by sexes^6^. Such spatial variation of unrecruited cases has been rarely reported in the literature but is critical to the planned geospatial analyses of environmental risk factors.

The key in the statistical estimation process we developed is the identification of *reference counties*. We employed three methods for this identification and the final selection of *reference counties* incorporates information from all three. While the three methods are based on quite different principles (z-score, straight section in a series, and Jenk’s natural breaks), their results well matched each other, which corroborates our methods for identifying *reference counties* in this study.

The literature on spatial smoothing for disease data is abundant. Most of those studies tackle statistical instability in observed disease rates, while some others use spatial smoothing to achieve imputation for missing data^21^. Different from most imputation studies, the spatial smoothing applied here targeted only those statistically abnormal areas. We considered such areas to be where the statistical estimation model, built to represent the ‘normal’ situation, would either overestimate or underestimate the incidence rate. Although the spatial adjustment in this study eventually had only a small effect on the statistical estimation results, we still included the spatial adjustment as the second phase in the entire estimation procedure because we consider it to be theoretically reasonable, and practically meaningful, and recommend that it be included in future similar studies.

Due to ALS being a rare disease, our estimation may suffer the “small number problem”, especially in those counties with small populations. More research is required to address and mitigate this problem, especially if additional confounding factors (e.g., age and other demographic variables) are to be incorporated and more precise geographic locations are to be investigated. Within the state of Ohio, confounding by age and sex distribution is likely minimal. However, these factors are critical to account for in other national or global scale contexts.

This section explains the reason we did not compare our current study with the traditional under-recruitment estimation method, capture-recapture. First, our data apparently does not meet the most basic assumption of the capture-recapture method: the data from each source are random samples of all patients. Second and more important, the capture-recapture method is a nonspatial method, i.e., it can only estimate the total number of unrecruited patients, but it cannot tell where those unrecruited patients are in the study area. On the other hand, our method can give the spatial distribution of those patients. For this second reason, even if we put the results of the total number estimated by the two methods together, we have no way to claim which one is better, as we do not know the true value for the total number. Our verification in this study is to evaluate the consistency between spatial distributions of the ALS mortality cases and the result of our estimation, which cannot be applied to the capture-recapture result.

The estimation method presented in this paper is novel and can be of use in studies of other regions and diseases that experience similar problems of incomplete case ascertainment. However, this current study has several limitations that hopefully can be addressed in future research: 1) While the selection of the reference county greatly influences the estimation, in this study, this selection was simply based on the association between ALS case counts and population. Given the increasing understanding of ALS etiology and its association with other factors, e.g., population density (urbanity and rurality), demographics, socioeconomics, and environment, we may be able to improve the selection of reference counties by considering the etiology and more relevant factors. 2) Inherently, the proposed method may overestimate the number of patients in a cold spot, i.e., an area whose disease rate is indeed lower than expected, due to certain local protective factors. Fortunately, such overestimation would not adversely impact research focusing on hotspots and risk factors. 3) While the ALS mortality data of Ohio is the best available data for us to verify the results of our method, such data entails considerable uncertainty when being used for such a purpose, especially when the verification is through a comparison of the spatial distributions of the two. Spatially, the location information in the mortality data is about where the person deceased, which is not necessary to be where the person had resided for long and/or where the person had been diagnosed. Temporally, there would be fluctuations in incidence and the mortality data may not fully reflect the situation of contemporary patients. After all, the use of mortality data is an indirect way of verification of patients. As the data collection of our research is still ongoing, hopefully, we should be able to use a more direct way to validate our estimation.

In summary, using the Ohio Registry of Amyotrophic Lateral Sclerosis as a case study, this study proposed a new method to estimate the number of cases not recruited by a patient Registry and their spatial distribution. The basic idea of this method is to borrow the data from the counties with relatively reasonable and reliable case counts (*reference counties*) to estimate the missing cases in other counties, and then locally smooth outliers. Here, we used three methods to identify the reference counties, which also could be further developed with more environmental information considered. The advantage of this new method in the spatial perspective makes it an alternative to capture-recapture for estimating cases missed by disease registries.

## Methods

### Recruited cases

**Figure 3:**
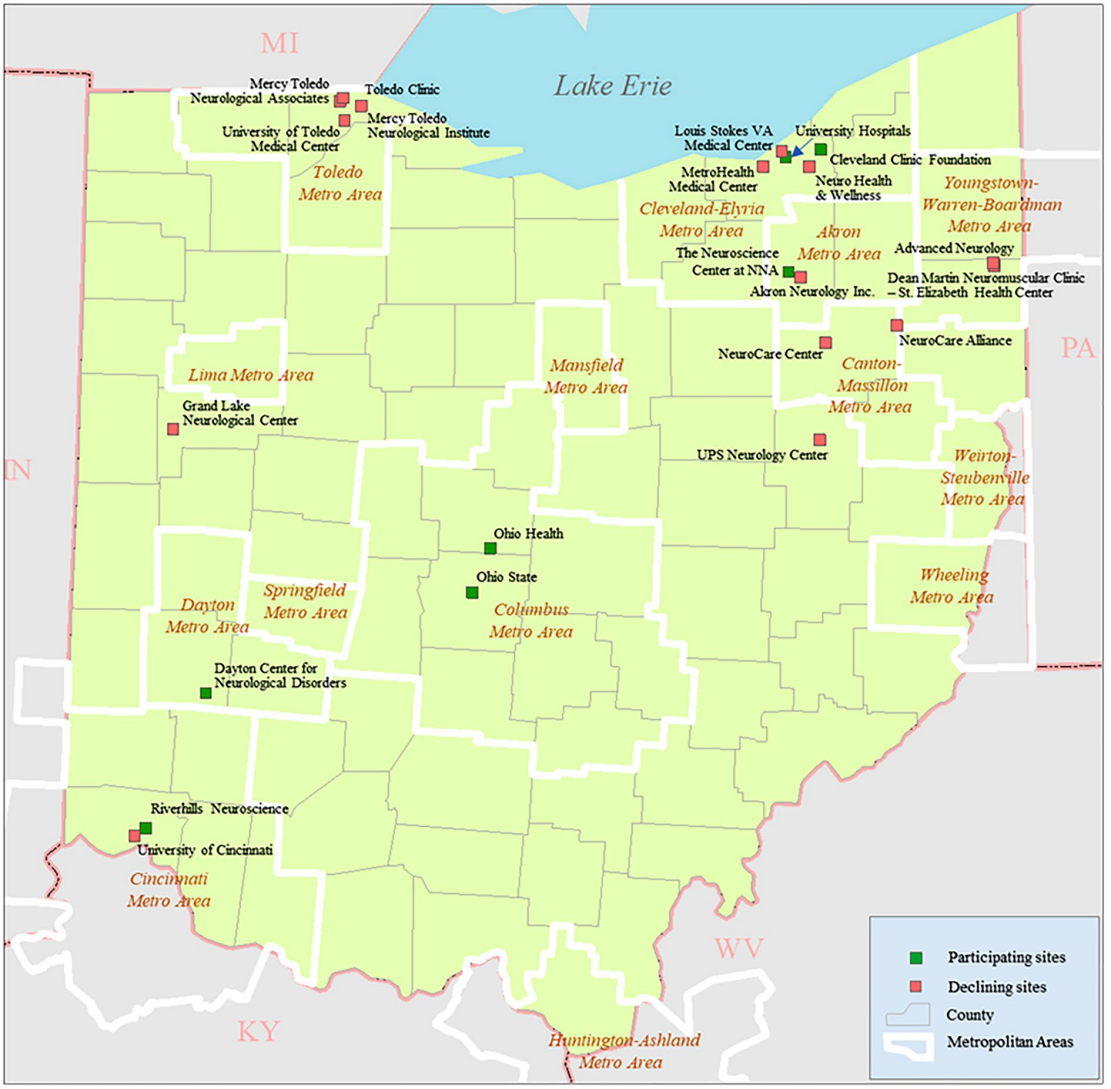
Sites are invited to participate in the Ohio ALS Registry. *Participating sites* are institutions and neurologists that have contributed cases to the Registry; *Declining sites* are those that have not.

The Ohio ALS Registry sought to count every patient newly diagnosed with ALS living in Ohio during the two years of October 1, 2016, through September 30, 2018. The clinical base of the registry was the Section of ALS and Related Disorders, Cleveland Clinic, Cleveland, OH^22^. We identified neurologists and referral centers that diagnose ALS throughout the state and invited them to participate in the Registry by submitting case-report forms, but we were not able to recruit all centers seeing ALS patients in Ohio (Fig. 3). A detailed description of the diagnostic standards employed, the case-recruiting process implemented, and case information collected has been published by Andrew et al.^6^. That study was approved by the Dartmouth-Hitchcock Medical Center IRB and by the IRB of institutions that did not defer to the Dartmouth-Hitchcock Medical Center IRB.

For each recruited case, we have collected the patient’s age and sex. For the present study, we have aggregated the patient’s location information to the county level, according to the patient’s residence at the time of diagnosis. We eliminated duplicate records of cases registered at more than one facility.

### Population data

We obtained population data from the Ohio Public Health Information Warehouse^23^, made available by the Ohio Department of Health. The data originated from the Vintage 2019 Bridged-Race Postcensal Population Estimates of the National Center for Health Statistics (NCHS)^24^. From the dataset, we extracted the county-level data for 2016 to use in this study.

### Mortality data

The mortality data we used to validate the estimated unrecruited ALS cases were from the State of Ohio Bureau of Vital Statistics^25^. We extracted vital records for the years 2016–2019 with ICD 10 codes G122 and G128 listed as the underlying or contributing cause of death. We determined the prior addresses of each deceased subject using a commercial marketing database, LexisNexis (Dayton, Ohio), which has residential records dating back to the mid-1990s. We used an estimated 4-year median survival time for ALS patients to assign the probable county of residence at the time of diagnosis to each deceased case^26,27^.

### Analysis design

Our method for estimating the spatial distribution of unrecruited ALS cases comprises two phases: statistical modeling and spatial adjustment. First, we statistically identified counties in Ohio with normal case-population relationships, with which we built a Poisson regression model and used it to run estimations for each county potentially having unrecruited cases. Second, following the principle of spatial autocorrelation^28,29^, we conducted spatial smoothing to adjust the results from the first phase.

Ideally, we would incorporate information about both age and sex, as they are associated with the ALS risk^27,30,31^. However, ALS is a rare disease and Ohio’s county-level case count in an age-sex decade was often too small to support a solid statistical analysis. Therefore, we only incorporated sex as a covariate in this study considering that Ohio has relatively homogeneous geospatial distributions of people of different ages within the state. The entire procedure, as described in the following subsections, was applied to female and male cases separately. As all the analyses in this study were at the county level in Ohio, it means all the data used in this study were anonymized and aggregated to each county of Ohio. It was not required to have any administrative permissions to access the data directly used in the current study.

### Statistical modeling

#### Identify reference counties

We intended to establish a model of the relationship between the *expected* case count and the population in each county of Ohio. We would then apply that model to each county that may have unrecruited cases to estimate its *expected* case count. The difference between the *expected* case count and the number of *recruited* cases would be the number of *unrecruited* cases in that county. To establish such a model, we needed to identify counties in Ohio whose incidence rates were within a normal range. Herein, we refer to such counties as *reference counties*.

To identify *reference counties*, we ranked all counties in descending order according to their incidence rates calculated with the recruited cases. Conceptually, we assumed that the *reference counties* should be chosen from the middle section of this ranked list, because those counties in the upper section may be in hotspot regions, whereas those in the lower section (*target counties*) may either have unrecruited cases or be in cold-spot regions.

In this study, we used a data exploratory approach to determine reasonable boundaries in the ranked list for identifying *reference counties*. We employed three different methods to achieve that determination. First, we considered that the incidence rate of *reference counties* should fall within a certain range around the mean incidence rate of all counties. We quantified that *certain range* by the number of standard deviations, i.e., the z-score in statistics (Table 4).

**Table 4:** ALS incidence rate ranges for determining *reference counties* in Ohio, as defined by the z-score.

Sex	Mean	Std. dev	Incidence rate range		
			|z|< 0.25	|z|< 0.5	|z|< 0.75
Female	3.1345	4.3187	2.7928–4.1869	1.5490–5.0802	0–6.3371
Male	5.2285	6.3039	3.6893–6.4969	2.3656–8.3850	0.5062–9.9020

**Figure 4:**
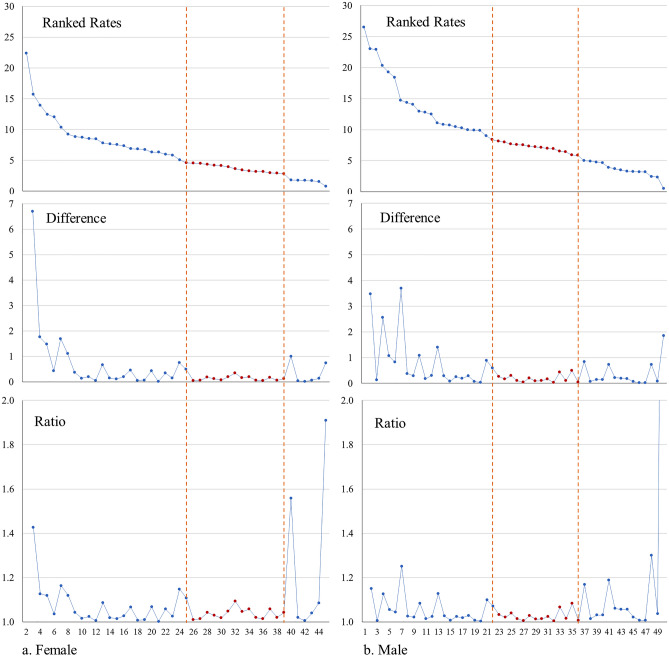
Plots of the ranked ALS incidence and the difference and ratio between two consecutive values. All plots are for the counties with non-zero incidence rates in Ohio.

Second, when we plotted the ranked list (Fig. 4), we observed a relatively straight section in the middle of both the male and female rate series. We presumed that the incidence rate of a *reference county* must lie within this section, as the rate values in this section were not extreme and less variable. We then calculated the difference and ratio between two consecutive rate values in the ranking sequence (Fig. 4). The boundaries of the straight section in the incidence series were then determined through the visual inspection of the flatness and smoothness in the difference series and the ratio series.

Third, we calculated Jenk’s natural breaks^32^, which divides a series of values into a prespecified number of sections in a way that minimizes the intra-section variance and maximizes the inter-section variance. Table 5 shows the sections defined using this method.

**Table 5:** Sections of the ranked ALS incidence rates in Ohio defined by Jenk’s natural breaks.

Sex	Section 1	Section 2	Section 3	Section 4	Section 5
Female	0–1.7920	2.7928–5.0802	5.8331–10.3416	12.0337–22.4039	N/A
Male	0–0.5062	2.3656–5.0181	5.8634–8.3850	8.9791–14.7384	18.4417–26.5113

For females, the specified number of sections = 4, and for males, the number = 5. N/A = not applicable.

The results of these three methods for identifying the reference counties (z-score, straight section, and Jenk’s natural breaks) were relatively consistent. For the female cases, the lower boundary of the straight section (2.7928) was the same as the lower boundary of the range defined by |z-score|< 0.25 and the lower boundary of section 2 defined by Jenk’s natural breaks, whereas the upper boundary of the straight section (4.5895) is only one county below the upper boundary shared by the range of |z-score|< 0.5 and section 2 defined by Jenk’s natural breaks (5.0802). For the male cases, the lower boundary of the straight section (5.8634) was at the county immediately above the mean value (there is no county exactly on the mean value) and the same as the lower boundary of section 3 of Jenk’s natural breaks, whereas the upper boundary of the straight section (8.3850) exactly corresponds to the upper boundary of |z-score|< 0.5 and the upper boundary of section 3 of Jenk’s natural breaks. We selected the final *reference counties* for males and females based on the results of all three methods, and each set happens to include 15 counties (red dots in Fig. 4 and Table 6).

**Table 6:** The final determining ranges, in terms of the incidence rate, for identifying *reference counties*.

Sex	Range of incidence rate for identifying *reference counties*	Number of identified *reference counties*
Female	2.7928–4.5895	15
Male	5.8634–8.3850	15

We used ArcGIS 10.7 to calculate Jenk’s natural breaks and Microsoft Excel for all other tabular calculations and plotting operations.

#### Build Poisson regression models

Since ALS is a rare disease and most *reference counties* have only one or two cases, we chose to use *Poisson regression* to build the model. The response variable was the case count of each county, and the explanatory variable was the natural logarithm of the county’s base population. The use of the natural logarithm of the population is to approximate a linear relationship in the modeling, as Poisson regression uses a logarithm link function. In this study, the Poisson regression was conducted using the package “glm” in R software. The output from the R operations is summarized in Table 7.

**Table 7:** Poisson regression models of county-level relationships between case count and population, built for *reference counties*.

		Female (N = 15)	Male (N = 15)
ln(female population)	Coefficient	1.0525	0.9524
	Std. error	0.1526	0.1208
	Z value	6.8950	7.8850
	Pr( >|z|)	0.0000	0.0000
Evaluation	Null deviance	42.2857 on 14 df	70.3104 on 14 df
	Residual deviance	0.7952 on 13 df	0.4607 on 13 df
	AIC	42.233	46.901

*df* degrees of freedom.

We used a Monte Carlo process to determine the 95% confidence interval (CI) for the estimates. For the sex-specific estimation of each county, we used the prediction and the standard error of each county output by the “gml” model in R to estimate the empirical normal distribution and then generated 10,000 random values from this distribution. The empirical 95% CI was estimated based on the 2.5^th^ and 97.5^th^ percentiles of the 10,000 results from this process. The sex-specific values were also used to estimate the 95% CI of the total value.

### Spatial adjustment

**Figure 5:**
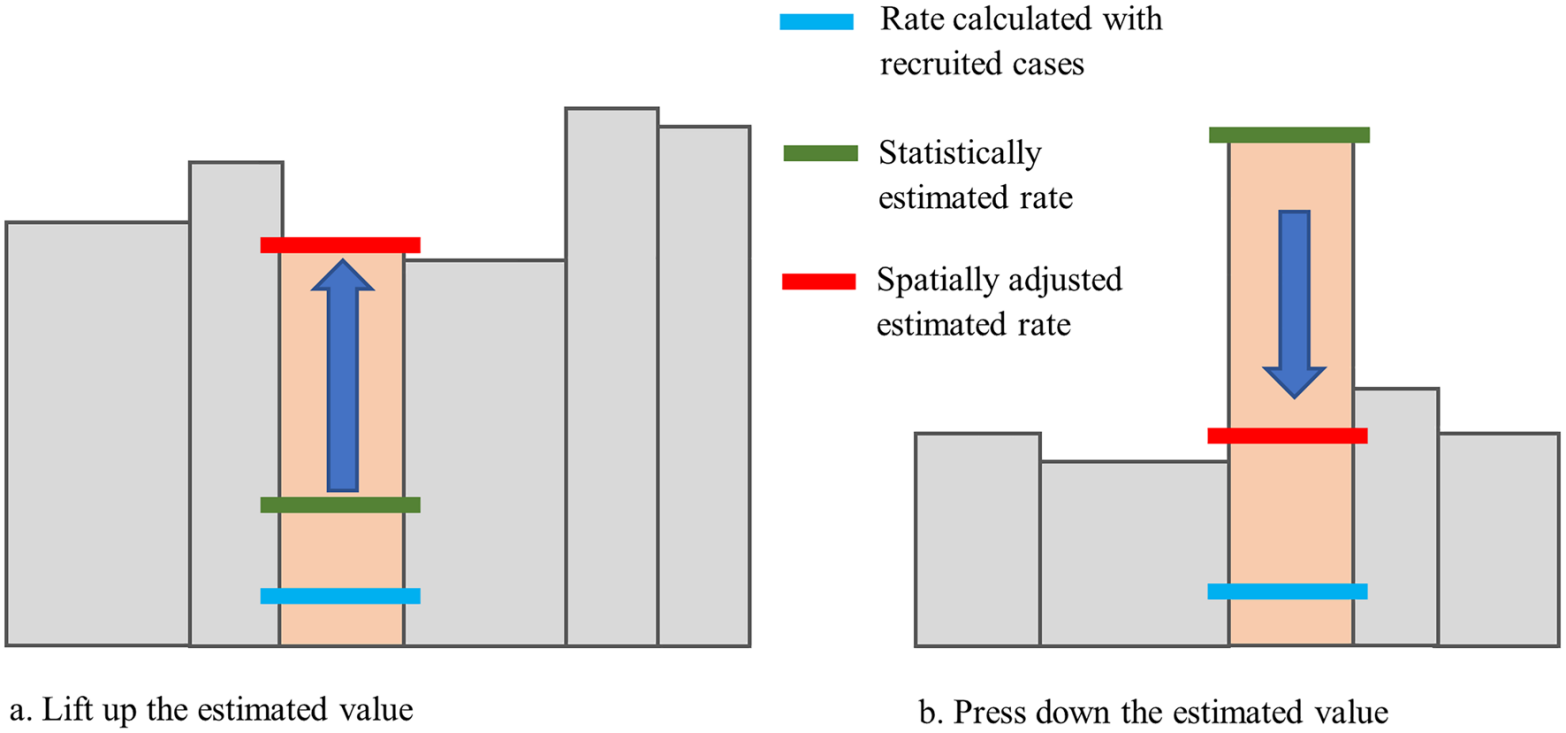
Spatial smoothing for adjusting the statistically estimated case counts in hotspot and cold spot areas.

The statistical estimation described above lifts the case count of a *target county* to a normal range, using assumptions based on the *reference county* values. However, if a *target county* with an unexpected low case count is in a hotspot or a *target county* with an unexpected high case count in a cold spot region, its actual case count is likely to deviate from the normal range and, thus, its estimated *expected* case count should be adjusted. This adjustment follows the principle of spatial autocorrelation^28,29^, i.e., nearby locations tend to have similar attribute values. The spatial autocorrelation is realistic in our case, as when an environmental factor causes a local abnormality in ALS risk, it will likely affect all counties in that region, rather than leaving one county out. Our spatial adjustment is based on this understanding and is illustrated in Fig. 5.

The spatial adjustment contains two steps. First, we identified local anomalies using the local Moran’s I analysis^33^, implemented with the *Local* *Moran’s I with EB Rate* tool in GeoDa^34^. The tool outputs indications of four types of local anomalies: clusters of high values (*HH*); clusters of low values (*LL*); outliers as a high value surrounded by low values (*HL*), and outliers as a low value surrounded by high values (*LH*). The tool reports statistical significance at *P* value = 0.01, 0.02, and 0.05. In this study, we focused on outlier *target counties*, labeled as *HL* or *LH*, and significance at *P* value = 0.05. The *Local* *Moran’s I with EB Rate* tool performs an Empirical Bayesian (EB) smoothing to the raw rate before calculating Moran’s I, addressing the small number problem due to a small case count and/or a small population.

Second, for a *target county* labeled as an outlier (*HL* or *LH*), we ran the locally-weighted-averaging smoothing to adjust its value^35,36^. This process calculates a weighted average of the values of all counties in the neighborhood of the *target county* and replaces the original value of the *target* county with that average. In this study, we defined the neighborhood by contiguity, i.e., if a county shared a boundary with the *target county*, it was considered a neighbor of the *target county*. The weight of each involved county was its population. The eventual value sent into the smoothing process was the incidence rate of a county rather than its case count. The adjusted rate of the *target county* was then used to calculate the case count of that county and in turn the number of its unrecruited cases.

### Validation

Since the median survival time from onset to death of ALS cases is usually 2–4 years from the first appearance of symptoms^26,27,37,38^, we used mortality data as validation. We were able to obtain residential history data on the cases identified from the Ohio mortality data so that we could identify the location of the patient’s home in the year of diagnosis. Assuming the mortality rates of ALS cases in a particular county are fairly stable over time, the spatial distribution of the residence at the time of diagnosis of the deceased ALS cases in Ohio should closely follow the spatial distribution of the Registry incidence that we observed. The ALS mortality data allowed us to compare the two spatial distributions to validate our estimation. We calculated Pearson’s correlation coefficient between the two, using each county as a unit in the calculation.

## Data Availability

The datasets generated during and/or analyzed during the current study are available from the corresponding author upon reasonable request.

## Abbreviations

ALS: Amyotrophic lateral sclerosis
NCHS: National Center for Health Statistics
CI: Confidence interval
HH: Clusters of high values
LL: Clusters of low values
HL: Outliers as a high value surrounded by low values
LH: Outliers as a low value surrounded by high values
EB: Empirical Bayesian
VA: Veterans Affairs
